# Spontaneous coronary artery dissection with concomitant vasospastic angina and false lumen enlargement: a case report

**DOI:** 10.1093/ehjcr/ytag417

**Published:** 2026-06-03

**Authors:** Yuka Oda, Takahiro Yamada, Ryuichi Usui, Akihiko Nogami, Kikuya Uno

**Affiliations:** Department of Cardiology, Institute of Medicine, University of Tsukuba, 1-1-1 Tennodai, Tsukuba 305-8575, Japan; Department of Cardiology, Tokyo Heart Rhythm Hospital, 3-25-17 Hirai, Edogawa-ku, Tokyo 132-0035, Japan; Department of Cardiology, Tokyo Women's Medical University Adachi Medical Center, 4-33-1, Kohoku, Adachi-ku, Tokyo 123-8558, Japan; Department of Cardiology, Tokyo Heart Rhythm Hospital, 3-25-17 Hirai, Edogawa-ku, Tokyo 132-0035, Japan; Department of Cardiology, Institute of Medicine, University of Tsukuba, 1-1-1 Tennodai, Tsukuba 305-8575, Japan; Department of Cardiology, Tokyo Heart Rhythm Hospital, 3-25-17 Hirai, Edogawa-ku, Tokyo 132-0035, Japan; Department of Cardiology, Tokyo Heart Rhythm Hospital, 3-25-17 Hirai, Edogawa-ku, Tokyo 132-0035, Japan

**Keywords:** Case report, Acute myocardial infarction, Spontaneous coronary artery dissection, Vasospastic angina, False lumen enlargement

## Abstract

**Background:**

Spontaneous coronary artery dissection (SCAD) is a rare cause of acute myocardial infarction (AMI). Although the exact pathophysiological mechanism of SCAD remains unclear, coronary vasospasm has been proposed as a possible associated or triggering factor.

**Case summary:**

A 39-year-old woman presented with sudden-onset back pain and ST-segment elevation in leads V1–V6 on electrocardiogram. Emergent coronary angiography (CAG) revealed subtotal occlusion with dissection in the mid left anterior descending artery (LAD), and intravascular ultrasound demonstrated that the dissection extended from the mid portion to the ostium of the LAD. We deployed a drug-eluting stent for the mid-LAD and confirmed reperfusion. After the PCI, she developed recurrent chest pain with transient ST-segment elevation in the inferior leads. Emergent CAG demonstrated diffuse severe stenosis in the distal right coronary artery. Both symptoms and electrocardiographic changes resolved spontaneously; coronary vasospasm was considered the most likely cause. On day 17, follow-up CAG revealed progression of stenosis from mild to severe in the mid to ostial LAD and a new dissecting lesion in the mid right coronary artery. Repeat PCI was performed, and the patient remained stable after discharge.

**Discussion:**

There are some SCAD cases that present acute progression or recurrence. The prognostic factor was unknown, while this case would suggest that vasospastic angina pectoris (VSA) might be associated with those events. Consideration of concomitant VSA may be warranted in the clinical management of patients with SCAD.

Learning points

- Vasospastic angina might coexist with spontaneous coronary artery dissection and appears to be associated with acute progression or recurrence.
- Consideration of concomitant vasospastic angina may be warranted in the clinical management of patients with spontaneous coronary artery dissection.

## Introduction

Spontaneous coronary artery dissection (SCAD) is a rare cause of acute myocardial infarction (AMI).^1^ However, it is the second most common cause among women aged 50 years or younger.^2^ SCAD is initiated by an intimal tear or medial disruption, leading to the formation of an intramural haematoma that compresses the true lumen, thereby impairing coronary blood flow and potentially causing AMI.^3^ Although the exact pathophysiological mechanism of SCAD remains unclear, coronary vasospasm has been proposed as a possible associated or triggering factor.

Here, we report a case of SCAD complicated with vasospastic angina pectoris (VSA), with progressive dissection in the acute phase presumably caused by multiple vasospasms.

## Summary figure

**Figure:**
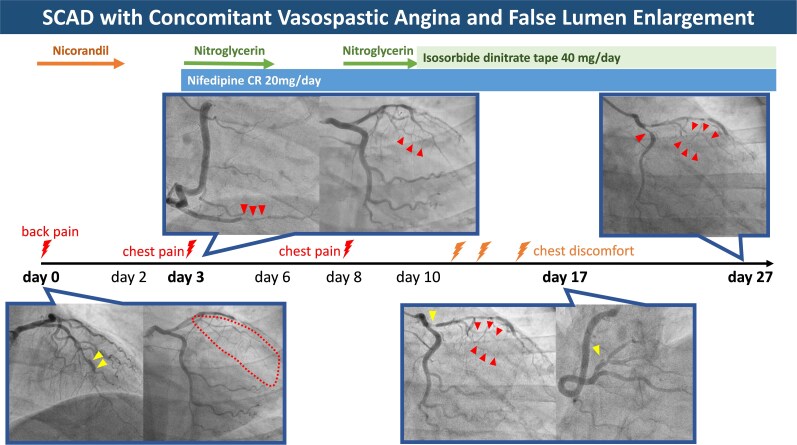


## Case presentation

A 39-year-old Japanese woman with no prior episodes of chest or back pain presented with sudden-onset back pain. Electrocardiogram (ECG) showed ST-segment elevation in leads V1–V6, and she was transferred to our hospital from a referring physician with suspected ST-elevation myocardial infarction (STEMI). On arrival, her back pain persisted, and her ECG also revealed ST-segment elevation in leads V1–V6 (*Figure 1A*). Transthoracic echocardiography demonstrated severe hypokinesis of the anterior wall. Based on these findings, anterior STEMI was diagnosed, and emergent coronary angiography (CAG) was performed. CAG revealed subtotal occlusion with dissection in the mid portion of the left anterior descending artery (LAD), suggesting spontaneous coronary artery dissection (SCAD) (*Figure 1B*, Supplementary material online, *Video S1*). In addition, diffuse spastic change was identified at the first diagonal branch (D1) (*Figure 1B*). Considering the persistent ST-segment elevation and ongoing symptoms, we performed emergent percutaneous coronary intervention (PCI) for LAD subsequently. Intravascular ultrasound demonstrated dissection extending from the mid portion to the ostium of the LAD, although the entry site of the dissection was unclear (*Figure 1C* and *D*, Supplementary material online, *Video S2*). Firstly, we performed fenestration using a cutting balloon several times, although coronary flow remained limited. Finally, a drug-eluting stent was deployed in the mid LAD, resulting in satisfactory reperfusion (see Supplementary material online, *Video S3*). While IVUS revealed residual intramural haematoma at the distal edge of the stent (see Supplementary material online, *Video S4*), there was no progression of the false lumen and coronary flow was preserved. Therefore, we considered that the entry point had been adequately covered and that additional stenting was not required. Vasospasm at the D1 repeatedly worsened and resolved during the primary PCI. Ultimately, vasospasm persisted in one branch of the D1, resulting in a slow-flow phenomenon, whereas spasm in the other branch improved. (see Supplementary material online, *Video S3*).

**Figure 1:**
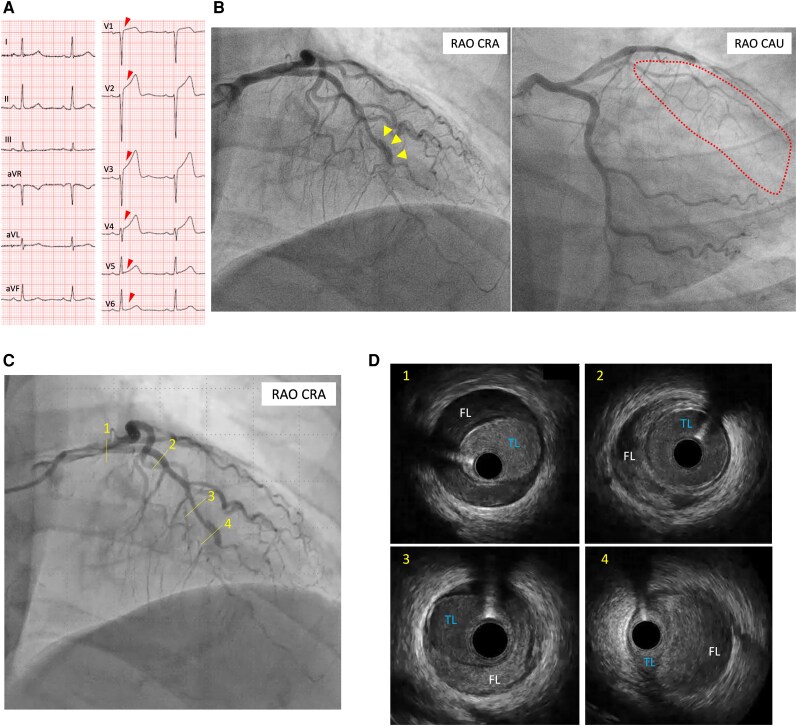
Twelve-lead electrocardiogram and emergent coronary angiography. (*A*) Twelve-lead electrocardiogram indicated ST-segment elevation in leads V1–V6 (triangles). No reciprocal changes were confirmed. (*B*) Left; Coronary angiography showed coronary artery dissection (triangles) in the mid left anterior descending artery (LAD) and subtotal occlusion. Right; The diffuse spastic stenosis was observed in the first diagonal branch (dot circle). (*C*) (*D*) Intravascular ultrasound (IVUS) revealed prolonged dissection from mid (4) to ostial LAD (1). The site of 2 is the proximal LAD, and the site of 4 is at bifurcation of third diagonal branch. The entry of dissection could not be identified by IVUS. CAU, caudal view; CRA, cranial view; FL, false lumen; RAO, right anterior oblique; TL, true lumen.

After the primary PCI, her menstruation began. Intravenous nicorandil was administered at 4 mg/h for two days. On day 3, the patient developed chest pain. Her electrocardiogram showed ST-segment elevation in leads II, III, and aVF (*Figure 2A*). Sublingual nitroglycerin failed to improve either the ST-segment elevation or the chest pain, prompting emergent repeat CAG. This revealed diffuse severe stenosis in the distal portion of the right coronary artery (RCA), without progression of the LAD dissection (*Figure 2B*, Supplementary material online, *Videos S5* and *S6*). Additionally, coronary flow in the D1 was restored, and no further spastic changes were observed. Because both the ST-segment elevation and symptoms resolved spontaneously, coronary vasospasm was considered the most likely cause. A calcium channel blocker (nifedipine CR, 20 mg/day) was initiated, and intravenous nitroglycerin was administered for two days.

**Figure 2:**
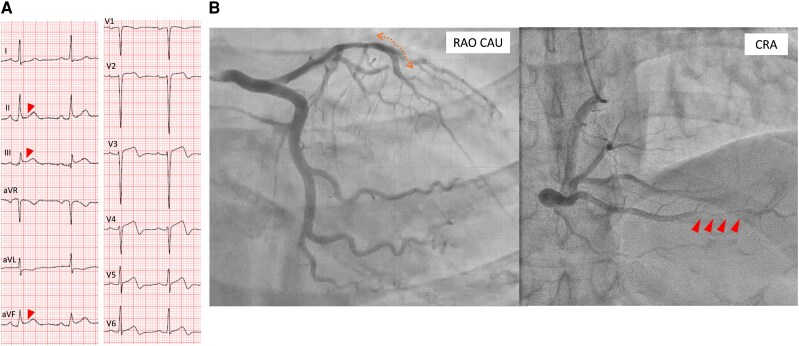
Twelve-lead electrocardiogram and emergent coronary angiography on day 3. (*A*) Twelve-lead electrocardiogram indicated ST-segment elevation in leads II, III and aVF (triangles). (*B*) Left; Coronary angiography showed no re-stenosis at the stent site (double-ended arrow). Right; Diffuse severe stenotic lesion suspected by vasospasm in the distal portion of the right coronary artery was observed (triangles). CAU, caudal view; CRA, cranial view.

After discontinuation of the nitroglycerin infusion, chest pain recurred. Therefore, an isosorbide dinitrate transdermal patch (40 mg/day) was added, resulting in symptom relief. However, the patient occasionally complained of atypical chest discomfort during rehabilitation. Consequently, repeat CAG was performed before discharge for reassessment. This demonstrated progression of the LAD dissection, particularly at the ostium, as well as a small dissection in the distal RCA (*Figure 3A* and *B*, Supplementary material online, *Videos S7* and *S8*). Furthermore, recurrent diffuse vasospasm was observed at the D1. After careful discussion of the optimal management strategy, including coronary artery bypass grafting, PCI was ultimately selected. On day 27, PCI was performed from the left main trunk to the LAD. At first, the ostial left circumflex and D1 indicated spastic stenosis (*Figure 4A*) and improved by infusion of nicorandil (see Supplementary material online, *Video S9*, Supplementary material online, *Figure S1*). Intravascular ultrasound revealed progressive severe stenosis from the mid to ostial LAD, and the thrombotic false lumen in the distal site from stent implantation (*Figure 4A* and *B*, Supplementary material online, *Video S10*). Two drug-eluting stents were deployed from the left main trunk to the LAD, achieving adequate vessel expansion and restoration of coronary flow. The patient was discharged on day 32. Follow-up coronary computed tomography revealed occlusion of the false lumen and patency of the stents in the left main trunk to the LAD (*Figure 5*).

**Figure 3:**
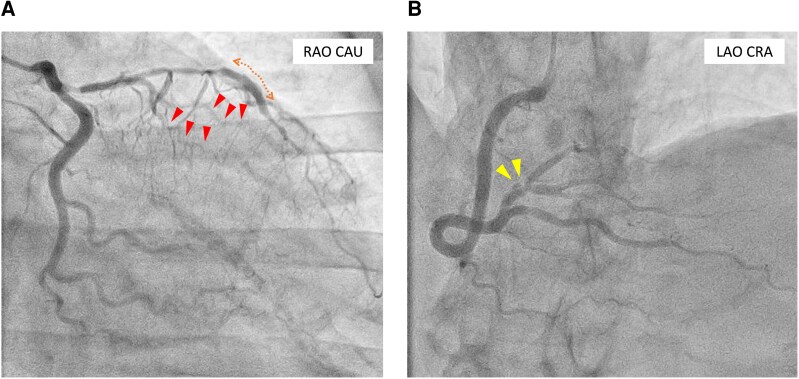
Follow-up coronary angiography on day 17. (*A*) Coronary angiography (CAG) indicated progression of stenosis from the mid to ostial left anterior descending artery (LAD). Ostial LAD stenosis has progressed to severe stenosis. A double-ended arrow indicates the site of stent implantation. The false lumen at the stent site was not thrombosed. The first diagonal branch showed diffuse spastic stenosis (triangles). (*B*) CAG also indicated the new dissecting lesion in the mid-right coronary artery (triangles). CAU, caudal view; CRA, cranial view; LAO, left anterior oblique; RAO, right anterior oblique.

**Figure 4:**
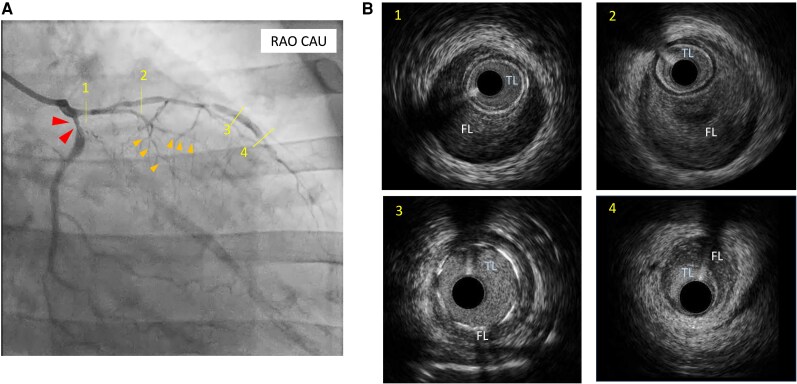
Percutaneous coronary intervention for the left main trunk to left anterior descending artery on day 27. (*A*) Coronary angiography (CAG) indicated more progression of stenosis in left anterior descending artery (LAD) and spastic change in the ostial left circumflex (large triangles). The first diagonal branch was severe diffuse spastic stenosis (small triangles). (*B*) Intravascular ultrasound also revealed severe stenosis from the mid (3) to ostial LAD (1). The thrombotic false lumen was observed in the distal site from stent implantation at the bifurcation of the third diagonal branch (4). The site of 2 is the proximal LAD, which also showed progression of dissection. CAU, caudal view; FL, false lumen; RAO, right anterior oblique; TL, true lumen.

**Figure 5:**
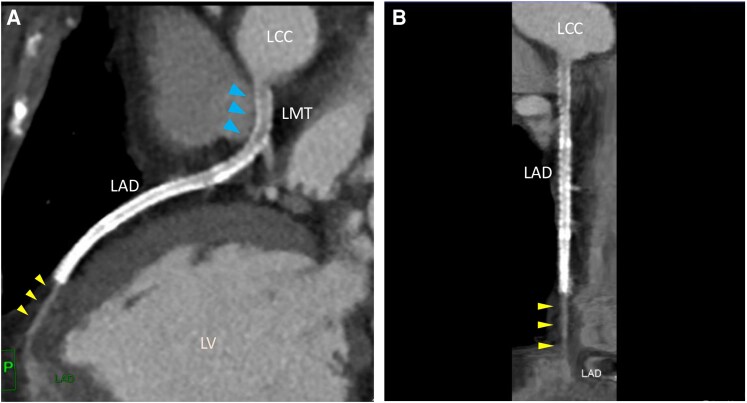
Coronary computed tomography after discharge. Coronary computed tomography confirmed no stent restenosis and thrombotic false lumen in the stent site of the left main trunk (large triangles). The distal site of stent implantation in left anterior descending artery was also patent (small triangles). LAD, left anterior descending artery; LCC, left coronary cusp; LMT, left main trunk; LV, left ventricle.

## Discussion

SCAD has been reported to coexist with coronary vasospasm,^4^ and previous case reports have suggested a possible association between vasospasm events and the development or recurrence of SCAD in the early phase.^5,6^ Although the true prevalence of this coexistence remains unclear, a single-center study in which provocative testing was performed in 10 patients with SCAD reported a positive response in 1 patient.^4^ SCAD is generally managed conservatively; however, a subset of cases may demonstrate progression requiring revascularization therapy. It has also been suggested that lesions treated with PCI are more likely to show further progression.^1^ Among such progressive cases, there might be some cases with coexisting VSA. In such progressive cases, prescription of beta-blockers that were beneficial for the prevention of recurrence of SCAD should be carefully considered because they might cause vasospasm. In the present case, a beta-blocker was initiated before diagnosing VSA. Fortunately, no overt VSA episodes were observed at initiation or dose escalation. However, a potential contribution to silent progression cannot be excluded. In addition, if such progressive cases coexist with hypertension, calcium-channel blockers should be considered over other antihypertensive drugs. Furthermore, the potential contribution of dual antiplatelet therapy to false lumen or intramural haematoma progression cannot be excluded, and the indication for PCI should be carefully considered.

In the present case, a provocation test was not performed, considering the potential risk of progressive retrograde dissection from the LAD to the left main trunk associated with induced vasospasm. Accordingly, the present case did not satisfy the diagnostic criteria for VSA, and there was no clear evidence of vasospasm involving LAD. However, during the clinical course, an episode suggestive of VSA, vasospasm in the distal site of RCA with the ST-segment elevation in inferior leads was observed on day 3. Additional spasm-related stenosis was noted in the proximal left circumflex on day 27, and repetitive spastic change and resolution were observed in the D1. Moreover, follow-up CAG on day 17 indicated a new dissecting lesion in the mid RCA. Given the apparent multivessel involvement, the absence of angiographically documented vasospasm in the LAD does not preclude the possibility of subclinical or transient spasm. Furthermore, the patient reported recurrent episodes of atypical chest discomfort during the rehabilitation. While these symptoms may represent post-SCAD-related chest discomfort, the possibility of recurrent minor vasospastic episodes cannot be entirely excluded. Another potential factor contributing to SCAD recurrence in the present case may be hormonal influences. The patient experienced the onset of menstruation on the day of admission, corresponding to a low-estrogen phase. Given that SCAD has been reported to occur more frequently during the menstrual period, the repeated episodes of vasospasm during this phase may have contributed to the recurrence of SCAD in this patient.

## Conclusions

Although SCAD recurrence and progression are generally considered uncommon, vasospasm may be one of the contributing mechanisms in selected cases. Consideration of concomitant VSA may be warranted in the clinical management of patients with SCAD.

## Supplementary Material

ytag417_Supplementary_Data

## Data Availability

The data supporting the findings of this case report are available from the corresponding author upon reasonable request.

## Lead author biography

Dr. Yuka Oda is a cardiologist at Tokyo Heart Rhythm Hospital, Japan. Her primary clinical specialty is catheter ablation for cardiac arrhythmias. In addition to electrophysiology, she is actively involved in the comprehensive care of patients with cardiovascular diseases. Her clinical interests include arrhythmia management as well as the diagnosis and treatment of complex coronary conditions.

## Notes

**Consent:** The authors confirm that written informed consent was obtained from the patient for the publication of this case report and any accompanying images.
