# Supplementary material for: Spontaneous coronary artery dissection with concomitant vasospastic angina and false lumen enlargement: a case report
Source: Eur Heart J Case Rep. 2026 Jun 3;10(6):ytag417. doi: 10.1093/ehjcr/ytag417 (PMC13308963; doi:10.1093/ehjcr/ytag417)
Supplement: ytag417_Supplementary_Data [file ytag417_supplementary_data.zip › Supplemental_R1.docx]

**Supplemental material**


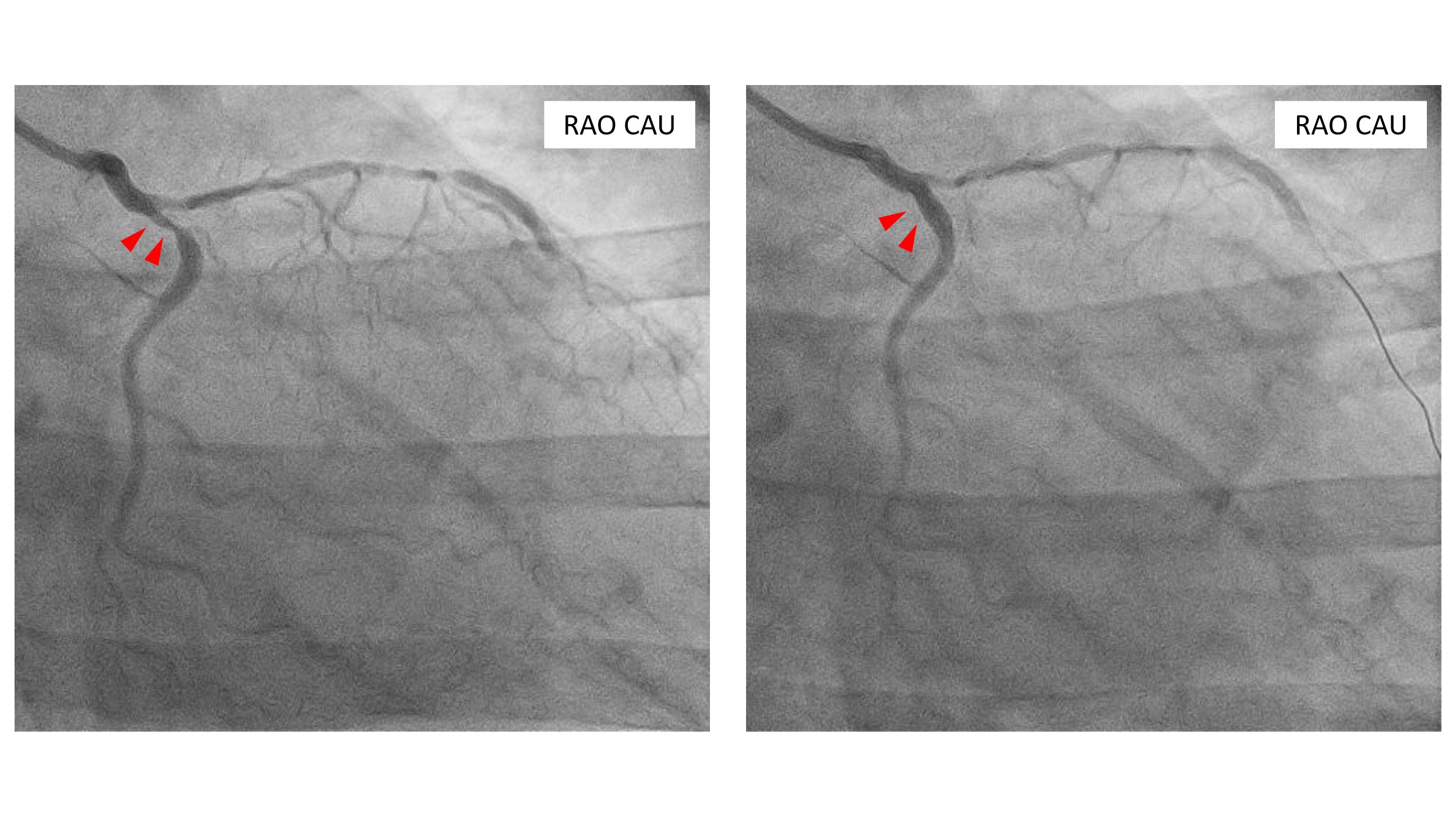
**Supplemental Figure S1. Coronary angiography on day27.**

Left. At the beginning of the procedure, stenosis at the ostium of left circumflex was observed.

Right. Following infusion of nicorandil, the stenosis improved, suggesting vasospasm.

CAU, caudal view; RAO, right anterior oblique.
